# Acute benefits of the microbial-derived isoflavone metabolite equol on arterial stiffness in men prospectively recruited according to equol producer phenotype: a double-blind randomized controlled trial^1^,^2^

**DOI:** 10.3945/ajcn.115.125690

**Published:** 2016-02-03

**Authors:** Sara Hazim, Peter J Curtis, Manuel Y Schär, Luisa M Ostertag, Colin D Kay, Anne-Marie Minihane, Aedín Cassidy

**Affiliations:** Department of Nutrition, Norwich Medical School, University of East Anglia, Norwich, United Kingdom

**Keywords:** CVD risk, isoflavone, flavonoids, vascular function, equol producer phenotype, arterial stiffness

## Abstract

**Background:** There is much speculation with regard to the potential cardioprotective benefits of equol, a microbial-derived metabolite of the isoflavone daidzein, which is produced in the large intestine after soy intake in 30% of Western populations. Although cross-sectional and retrospective data support favorable associations between the equol producer (EP) phenotype and cardiometabolic health, few studies have prospectively recruited EPs to confirm this association.

**Objective:** The aim was to determine whether the acute vascular benefits of isoflavones differ according to EP phenotype and subsequently investigate the effect of providing commercially produced S-(–)equol to non-EPs.

**Design:** We prospectively recruited male EPs and non-EPs (*n* = 14/group) at moderate cardiovascular risk into a double-blind, placebo-controlled crossover study to examine the acute effects of soy isoflavones (80-mg aglycone equivalents) on arterial stiffness [carotid-femoral pulse-wave velocity (cfPWV)], blood pressure, endothelial function (measured by using the EndoPAT 2000; Itamar Medical), and nitric oxide at baseline (0 h) and 6 and 24 h after intake. In a separate assessment, non-EPs consumed 40 mg S-(–)equol with identical vascular measurements performed 2 h after intake.

**Results:** After soy intake, cfPWV significantly improved in EPs at 24 h (cfPWV change from 0 h: isoflavone, −0.2 ± 0.2 m/s; placebo, 0.6 ± 0.2 m/s; *P* < 0.01), which was significantly associated with plasma equol concentrations (*R* = −0.36, *P* = 0.01). No vascular effects were observed in EPs at 6 h or in non-EPs at any time point. Similarly, no benefit of commercially produced S-(–)equol was observed in non-EPs despite mean plasma equol concentrations reaching 3.2 μmol/L.

**Conclusions:** Acute soy intake improved cfPWV in EPs, equating to an 11–12% reduced risk of cardiovascular disease if sustained. However, a single dose of commercially produced equol had no cardiovascular benefits in non-EPs. These data suggest that the EP phenotype is critical in unlocking the vascular benefits of equol in men, and long-term trials should focus on confirming the implications of EP phenotype on cardiovascular health. This trial was registered at clinicaltrials.gov as NCT01530893.

## INTRODUCTION

There is growing interest in the cardioprotective role of the microbial-derived isoflavone metabolite equol, which is produced by 20–30% of Western (1) and 50–60% of Asian (2) populations after consumption of daidzein-rich soy foods. This has largely been stimulated by favorable associations in cross-sectional analysis between the equol producer (EP)^4^ phenotype and established markers of vascular function, including blood pressure (BP) (3), arterial stiffness, and endothelial function (4) and, likewise, biomarkers relevant to vascular health including lipids (3, 5), inflammatory biomarkers, and nitric oxide (NO) (3, 6, 7). In support of these associations, pharmacokinetic studies have indicated that equol has a higher systemic bioavailability and slower elimination rate (8, 9), and in vitro studies have reported greater antioxidant (10, 11) and vasodilator capacities than its precursor, daidzein (12, 13). Retrospective analysis of isoflavone intervention studies [lasting between 4 wk (14) and 1 y (7, 15)] also identified that the EP phenotype was associated with improvements in BP (4, 7, 15), arterial stiffness (7), and endothelial function (14). However, to date, few trials have recruited men or have been designed to prospectively determine the importance of the EP phenotype on vascular function by recruiting on the basis of EP phenotype. As a consequence, the ability to robustly interpret the importance of EP phenotype, or equol per se, remains unclear. In consideration of the vascular disease risk in men (16), studies assessing the importance of the EP in men should also be a focus.

In the 2 previous studies that prospectively recruited female postmenopausal EPs and provided isoflavone interventions lasting 8 wk (4) and 6 mo (17), the results contradicted the cross-sectional and retrospective associations previously reported, with BP, arterial stiffness, and endothelial function being unaltered (4, 17). In both instances, however, the relevance of the results is difficult to interpret. In one of the studies (4) the synthetic steroid hormone drug tibolone was coadministered, which has previously been shown to influence endothelial function (18, 19), whereas the more recent study by Liu et al. (17) did not include a matched non-EP group to compare the relative efficacy and provided an active placebo control (milk), which may explain the improvements in BP and flow-mediated dilation after receiving the placebo (17). To date, the acute or longer-term vascular benefits of the EP phenotype are largely unknown.

An alternative approach to harness the potential vascular benefits of equol per se has also emerged, with the development of exogenously produced equol products, either chemically synthesized, or more recently produced through soy germ fermentation by an equol-producing lactic acid bacterium *Lactococcus* 20-92 (SE5-OH; described further in references 8 and 20). Recently, the effect of this commercial bacterium–produced S-(–)equol on markers of cardiovascular disease (CVD) risk were assessed, with reductions in glycated hemoglobin, LDL cholesterol, and arterial stiffness observed in postmenopausal non-EPs after daily intake of a 10-mg S-(–)equol supplement for 12 wk (21). Because pharmacokinetic data have previously shown that commercially produced S-(–)equol is rapidly absorbed, with peak plasma concentrations within 1–2 h after administration (8, 9), further investigations are required to establish the acute vascular response at these peak concentrations.

We therefore prospectively recruited on the basis of EP phenotype and identified nonmedicated men with moderate CVD risk (22). The study hypothesis was that acute vascular function was mediated by circulatory equol, especially endothelial function (our primary outcome). We compared the vascular response to a single dose of isoflavones in EPs and non-EPs matched for cardiovascular-related factors determined a priori, and subsequently investigated whether providing commercially produced S-(–)equol supplements to non-EPs resulted in vascular responses similar to those observed in EPs after consumption of a daidzein-rich supplement.

## METHODS

### Study population

Healthy men aged 50–75 y who were screened to be at a 10–20% 10-y absolute risk of CVD (22) were recruited by the research scientists and research nurses. Ineligibility criteria were as follows: history of smoking (recent past or present); a clinical diagnosis of vascular disease, diabetes, or cancer; hepatic, renal, digestive, hematologic, neurological, or thyroid disorders; a resting BP >160/95 mm Hg at screening; and prescribed antihypertensive, statin, or antibiotic medications.

**FIGURE 1:**
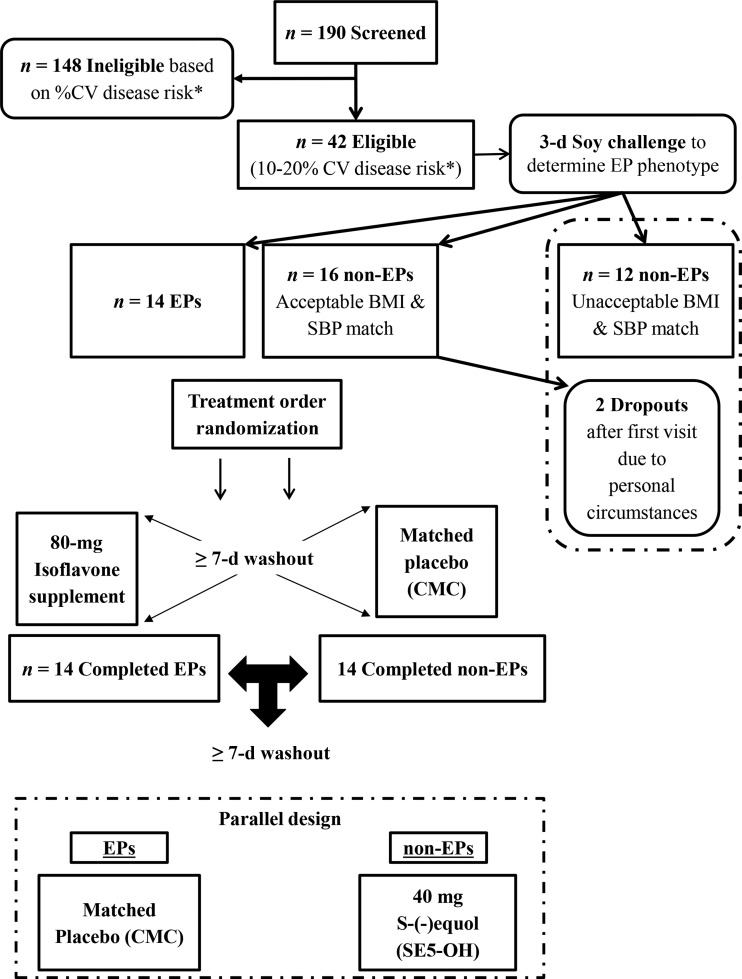
Enrollment, randomization, and trial design. *Ten-year absolute percentage of cardiovascular disease risk calculated at screening by using the British Hypertension Society risk calculator (22), incorporating plasma lipids, BMI, and SBP. CMC, carboxy-methyl cellulose; CV, cardiovascular; EP, equol producer; SBP, systolic blood pressure.

To prospectively recruit EPs, a soy challenge was undertaken according to standard methods (1); briefly, a commercially available daidzein-rich soy protein bar providing ∼160 mg soy isoflavones (aglycone equivalents), containing ∼64 mg daidzein (Revival Products), was consumed daily over 3 consecutive days, with urinary concentrations of equol and daidzein quantified by using validated liquid chromatography–tandem mass spectrometry (MS/MS) methods (23) from the first urine void on the fourth morning (**Figure 1**). EPs were defined as urinary log_10_ S-equol/daidzein ratio ≥−1.75, according to standard methods (1). Subsequently, an independent scientist matched 14 EPs with 14 non-EPs, with the groups balanced for factors considered likely a priori to affect vascular function, namely BMI and BP (Figure 1). The allocation to treatment order was randomly assigned by using a computer-generated, random-number sequence list.

The study was conducted at the Clinical Research and Trials Unit between April 2012 and August 2013 (University of East Anglia, United Kingdom) after National Research Ethics Committee approval. The study followed the principles of the Declaration of Helsinki and was registered at www.clinicaltrials.gov (NCT01530893). Participants gave their informed written consent before enrollment.

### Study design

The study was conducted by using a randomized, double-blind, placebo-controlled crossover design. Before each assessment visit, a number of dietary and lifestyle restrictions were implemented, including avoidance of flavonoid-containing supplements (for ≥1 mo before and during the study), avoidance of dietary flavonoids for 72 h (a list was provided), and avoidance of strenuous exercise (for 48 h), caffeine, and alcohol (for 24 h). Non–flavonoid-containing supplements (e.g., fish oils) were maintained at habitual intake amounts throughout the study. To control for the potential confounding effect on vascular function of nitrate intake on circulating nitrite (NO_2_^−^) and NO (24), participants were also instructed to avoid NO_2_^−^- and nitrate (NO_3_^−^)-rich foods (for 24 h; a list was provided) and to consume low NO_2_^−^- and NO_3_^−^-containing commercially available bottled water (Buxton; Nestlé Ltd.). A standardized pasta meal (Brake Bros Ltd.) was also provided, which was to be consumed on the evening before each assessment visit. Habitual dietary intakes were assessed by 24-h dietary recalls (assessed by using standardized food intake software; WISP version 3.0; Tinuviel).

Participants attended the clinical facility after a ≥10-h overnight fast, and then underwent a standardized sequence of vascular function assessments made at baseline and 6 and 24 h after the intervention. Timings were selected to coincide with peak plasma concentrations of isoflavones [i.e., daidzein and genistein peak at ∼6 h (25, 26)] and metabolites [including equol at ∼24 h (27)]. At each assessment time point, 15 min of supine rest in a standardized quiet, light- and temperature-monitored clinical room (21–24°C) was followed by BP measurements (taken in triplicate, each separated by 3 min) with the use of an automated sphygmomanometer Omron 705IT (Omron Health Care). Subsequently, carotid-femoral pulse-wave velocity (cfPWV), cardiac output, and augmentation index were assessed (Vicorder; Smart Medical), as described previously (references 28 and 29, respectively), and endothelial function (our primary outcome measure) was measured via peripheral arterial tonometry, using finger plethysmography, to produce a reactive hyperemia index score (EndoPAT 2000; Itamar Medical), as described previously (30). Blood samples were taken at the same time points (baseline and at 6 and 24 h), and plasma was stored at −80°C until subsequent concentration assessments of NO (NO_2_^−^ + NO_3_^−^) and circulating isoflavone metabolites. Participants were provided with a standardized lunch (cheese sandwiches and vanilla yogurt, providing 31 g protein, 96 g carbohydrate, and 27 g fat; 753 kcal) 2.5 h after consumption of the isoflavone or placebo capsules; no other food was provided before this meal. Participants consumed another standardized pasta meal and low-NO_2_^−^/NO_3_^−^ bottled water at home before returning in the fasted state to the facility for their 24-h assessment.

An identical sequence of vascular function assessments was made at the SE5-OH (commercial bacterium–produced equol) assessment visit at baseline and at 2 h after the intervention; the rationale for the shorter assessment was based on previous pharmacokinetic studies that showed maximum synthetic equol concentrations at 1–2 h after administration (8, 31).

### Administration of intervention products and treatment blinding

Intervention treatments were prepared by independent scientists and provided to participants as opaque capsules (held within opaque, tamper-evident bottles). A computer-generated, random-sequence allocation list was produced by an independent scientist and followed by the administrator of the treatments. The study scientists conducting the vascular assessments and analyzing the data remained blinded throughout the study.

### Intervention products for isoflavone assessment

At each assessment, 1 of 2 interventions was administered (following a randomly generated sequence, separated by ≥1 wk): *1*) an isoflavone capsule providing 80-mg aglycone equivalents of daidzein and genistein [a commercially available SoyLife extract (40%) with a typical soy germ ratio of genistein:daidzein:glycitein (15:50:35); Frutarom] or *2*) a carboxy-methyl cellulose–containing placebo capsule. A dose of 80 mg isoflavones was chosen, because this represents the midpoint dose from studies that previously showed improvements in endothelial function (26, 32) and that is achievable through a diet containing isoflavone-rich soy foods.

### Intervention products for commercially produced S-(–)equol assessment

To then determine the acute vascular effects of providing commercially produced, bacterium-derived S-(–)equol supplements (SE5-OH; Otsuka Pharmaceutical Co. Ltd.) to non-EPs, a further intervention arm was conducted: a 40-mg S-(–)equol dose was given to non-EPs and a placebo dose of carboxy-methyl cellulose (matched for weight, color, and number of capsules) was given as a control intervention to EPs. In previous studies, maximum concentrations of 1.42 μmol/L in men (31) and 4.95 μmol/L in postmenopausal women (8) were reported 1 h after the ingestion of 30 mg commercially produced equol (SE5-OH). We chose a 40-mg dose of S-equol, because it was previously shown to be bioavailable (33) and was considered likely to attain plasma concentrations a priori in men, which would be consistent with in vitro data showing NO-dependent relaxation in endothelium-intact aortic rings [at concentrations between 100 nmol and 10 μmol/L (34)] and a human intervention study that showed NO-dependent vasodilation in brachial arteries after the infusion of 3 μmol dehydroequol/L (35).

### Plasma isoflavone and NO analysis

Plasma isoflavones (daidzein, genistein, and glycitein) and the metabolite equol were analyzed by using previously published methods (23, 36). Briefly, plasma hydrolysis was performed by using β-glucuronidase and sulfatase in phosphate buffer (pH 5) with taxifolin as an internal standard; thereafter, the supernatant was extracted via solid-phase extraction (Starta-X tubes; Phenomenex; extraction efficiency of 81–92% over 3 replicates). Extracts of 200 μL were then analyzed by using an Agilent 1200 series HPLC (fitted with a Kinetex PFP column; Phenomonex) with a 3200 series Q-trap liquid chromatography–MS/MS system (Sciex) and multiple-reaction monitoring. Analytes were identified and quantified relative to pure standards by using precursor and product transitions. The HPLC-electrospray ionization-MS/MS methods were validated for linearity and precision with mean intra- and interassay precisions established as 12% and 11%, respectively.

Plasma samples were ultrafiltered (10 kDa) before duplicate assessment for total NO concentrations (determined as the total of NO_2_^−^ + NO_3_^−^) by using a commercial colorimetric assay (product 780001; Cayman Chemical Co.). Intra- and interassay CVs were 12.6% and 6.2%, respectively.

### Sample size and statistical analysis

To detect a 0.35 increase in reactive hyperemia index (our primary outcome measure) [assuming an SD of 0.4 on the basis of previous data (37) with 80% power and at the 5% significance level], a sample size of *n* = 14 participants/group was required to complete the study. Data are presented as means ± SEMs. Baseline participant characteristics (EPs compared with non-EPs) were assessed by using Student’s independent *t* tests, with the Shapiro-Wilk test (for normality) and Levene’s test (equality of variance for univariate analysis). At baseline, differences between the intervention groups in plasma concentrations and hemodynamic and vascular measures were evaluated by using ANOVA. The primary intervention analysis was “change from baseline” assessed by a mixed general linear model including interaction between 1 within-subject factor (repeated-measure ANOVA for treatment) and 1 between-subject factor (EP phenotype). All of the models were adjusted for age; BMI and treatment order were not significant and were excluded from the model. Bonferroni post hoc corrections were conducted where significant between-group differences were identified. The Spearman nonparametric test was used for bivariate correlations between plasma equol and vascular endpoints because plasma equol concentrations were not normally distributed. The treatment effects of the commercially produced S-(–)equol supplement was assessed via a general linear model (univariate analysis), which accounted for EP phenotype as a between-subject factor. Statistical analyses were performed by using SPSS software (version 22), and *P* values < 0.05 were considered significant.

## RESULTS

After a 3-d soy challenge, 33% of screened volunteers were categorized as EPs and were matched for BMI and BP with non-EPs (*n* = 14 EPs and *n* = 14 non-EPs completed the study; see Figure 1). In the non-EP group, 2 additionally enrolled participants discontinued the study (after 1 experimental period) due to a change in personal circumstances. No serious adverse events were reported. Across the completers in both groups, mean ± SEM age and BMI (in kg/m^2^) were 60 ± 1 y (range: 50–70 y) and 26.7 ± 0.7 (range: 18.7–35.4), respectively, and the mean British Hypertension Society algorithm–derived CVD risk was 15%, with no intergroup differences at baseline (**Table 1**).

**TABLE 1:** Participant characteristics at baseline for the male EPs and non-EPs^1^

	Non-EPs (*n* = 14)	EPs (*n* = 14)
Age,^2^ y	62 ± 2 (54–70)	57 ± 1 (50–69)
BMI, kg/m^2^	25 ± 1	26 ± 1
Height, m	1.77 ± 0.01	1.80 ± 0.02
LDL cholesterol, mmol/L	3.82 ± 0.20	3.86 ± 0.21
Triglycerides, mmol/L	1.24 ± 0.15	1.49 ± 0.20
Systolic BP, mm Hg	133 ± 2	131 ± 3
Diastolic BP, mm Hg	80 ± 1	81 ± 2
Ten-year CVD risk,^3^ %	16 ± 1	14 ± 1

1 Values are means ± SEMs unless otherwise indicated. All *P* > 0.05, except for age (*P* = 0.01) (Student’s independent *t* test). BP, blood pressure; CVD, cardiovascular disease; EP, equol producer.

2 Range in parentheses.

3 The 10-y absolute cardiovascular disease risk as calculated using the British Hypertension Society risk calculator (22).

**FIGURE 2:**
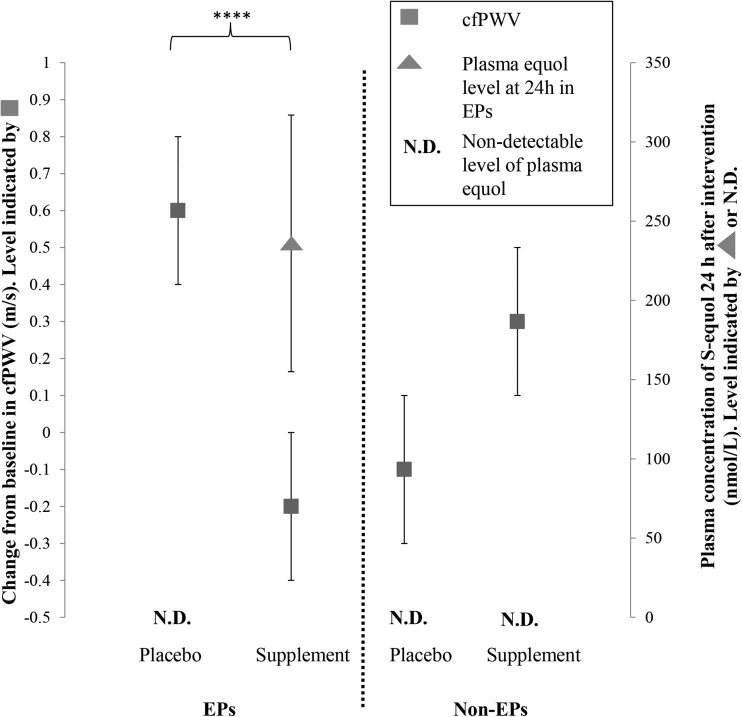
Acute effect of isoflavone consumption after 24 h on cfPWV in EPs and non-EPs. Values are means ± SEMs. Differences in study endpoints between interventions were analyzed by using ANOVA, with changes from baseline assessed by using a mixed general linear model including interaction between 1 within-subject factor (repeated-measures ANOVA for treatment) and 1 between-subject factor (EP phenotype). Bonferroni post hoc corrections were conducted where significant between-group differences were identified. Further analysis with the use of repeated-measures ANOVA for intervention effect (isoflavone compared with placebo in EPs for change from 0 to 24 h) was performed. ****P = 0.002 for cfPWV and *P* = 0.007 for S-equol. cfPWV, carotid-femoral pulse-wave velocity; EP, equol producer; N.D., not detectable.

cfPWV significantly decreased in EPs at 24 h after intake of an isoflavone-rich capsule compared with after placebo (cfPWV change from baseline: −0.2 ± 0.2 m/s after isoflavone and +0.6 ± 0.2 m/s after placebo; *P* = 0.002) (**Figure 2**). Notably, the highest plasma equol concentrations were attained in EPs at 24 h (*P* < 0.01) (**Table 2**), and equol concentrations were significantly correlated with changes in cfPWV (*R* = −0.36, *P* = 0.01). There were no cfPWV improvements for non-EPs at 24 h after isoflavone intake (Figure 2). Likewise, neither EPs nor non-EPs showed improved cfPWV values at 6 h (cfPWV change from baseline to +6 h—EPs: +0.5 ± 0.2 m/s after isoflavone and +0.2 ± 0.2 m/s after placebo; NS; non-EPs: −0.3 ± 0.2 m/s after isoflavone and −0.1 ± 0.2 m/s after placebo; NS).

**TABLE 2:** Plasma concentrations of isoflavones at baseline and 6 and 24 h postintervention in non-EPs and EPs after intake of placebo or isoflavone (80 mg isoflavone as aglycone equivalents)^1^

	Intervention, nmol/L							
	Placebo			Isoflavone supplement			*P*	
EP phenotype and isoflavone	0 h	6 h	24 h	0 h	6 h	24 h	6 h	24 h
Non-EPs								
Genistein	4 ± 3	15 ± 9	7 ± 5	4 ± 3	301 ± 40	72 ± 37	<0.0001	0.093
Daidzein	4 ± 2	16 ± 7	8 ± 4	4 ± 2	1692 ± 269	170 ± 46	<0.0001	0.002
S-equol	ND	ND	ND	ND	ND	ND	NA	NA
Glycitein	21 ± 7	20 ± 13	3 ± 2	21 ± 7	291 ± 48	54 ± 12	<0.0001	<0.0001
EPs								
Genistein	ND	ND	3 ± 3	ND	200 ± 33	28 ± 15	<0.0001	0.11
Daidzein	1 ± 1	3 ± 2	4 ± 2	1 ± 1	1688 ± 215	190 ± 31	<0.0001	<0.0001
S-equol	ND	ND	ND	ND	ND	236 ± 81	NA	0.007
Glycitein	19 ± 6	7 ± 4	6 ± 4	19 ± 6	237 ± 36	35 ± 9	<0.0001	0.009

1 Values are means ± SEMs; *n* = 14/group. *P* values represent the differences (by using ANOVA) in circulating concentrations of isoflavones and equol between the isoflavone supplement and placebo groups at 6 and 24 h after treatment. Isoflavone concentrations were significantly greater at 6 and 24 h after the isoflavone supplement in EPs and non-EPs (*P* < 0.05 for all, except for genistein at 24 h and glycitein at 24 h in EPs; see Supplemental Table 2). Conversely, there were no significant differences at either 6 or 24 h after consumption of placebo in EPs or non-EPs (*P* > 0.05 for all; see Supplemental Table 3). EP, equol producer; NA, not applicable; ND, nondetectable or below the limit of detection.

As expected, plasma concentrations of daidzein, genistein, and glycitein were significantly higher at 6 h after isoflavone intervention (all *P* < 0.01; non-EPs: 301 ± 40, 1692 ± 269, and 291 ± 48 nmol/L, respectively; EPs: 200 ± 33, 1688 ± 215, and 237 ± 36 nmol/L, respectively; Table 2); however, there were no other effects observed for either EPs or non-EPs across the range of vascular measures assessed at 6 or 24 h (**Table 3**). Likewise, total NO concentrations (NO_2_^−^ + NO_3_^−^) were not significantly different after isoflavone intake in EPs or non-EPs either at 6 or 24 h (total NO from baseline to 6 and 24 h after isoflavone intake—EPs: +0.02 ± 0.24 and −0.28 ± 0.28, respectively; both NS; non-EPs: +0.06 ± 0.24 and −0.05 ± 0.10, respectively; both NS).

**TABLE 3:** Hemodynamic and vascular measures at 0 h and change from baseline at 6 and 24 h after placebo and isoflavone intervention (80 mg isoflavones) in male EPs and non-EPs^1^

EP phenotype, treatment, and time	RHI	Diastolic BP, mm Hg	Systolic BP, mm Hg	CO, L/min	AI, %
EPs					
Placebo					
0 h	2.72 ± 0.16	82 ± 3	122 ± 6	3.92 ± 0.21	24 ± 1
0–6 h	−0.38 ± 0.23	−5 ± 2	−3 ± 3	0.54 ± 0.15	−3 ± 1
0–24 h	0.22 ± 0.20	3 ± 1	2 ± 3	0.31 ± 0.18	−2 ± 1
Isoflavones (80 mg)					
0 h	2.62 ± 0.17	83 ± 3	123 ± 6	4.09 ± 0.21	25 ± 1
0–6 h	−0.46 ± 0.27	−6 ± 2	−3 ± 3	0.56 ± 0.24	−3 ± 1
0–24 h	0.33 ± 0.18	0 ± 2	2 ± 3	0.11 ± 0.17	−3 ± 1
Non-EPs					
Placebo					
0 h	2.57 ± 0.15	76 ± 2	128 ± 2	4.30 ± 0.18	26 ± 2
0–6 h	0.39 ± 0.22	−4 ± 2	−2 ± 3	0.18 ± 0.15	−2 ± 1
0–24 h	0.05 ± 0.19	2 ± 1	1 ± 3	−0.06 ± 0.18	−1 ± 1
Isoflavones (80 mg)					
0 h	2.58 ± 0.15	76 ± 2	128 ± 3	4.32 ± 0.17	26 ± 1
0–6 h	−0.05 ± 0.26	−5 ± 2	−7 ± 3	0.03 ± 0.24	−2 ± 1
0–24 h	−0.07 ± 0.18	0 ± 2	2 ± 3	0.23 ± 0.17	−2 ± 1
*P*	0.55	0.56	0.99	0.08	0.95

1 Baseline values are means ± SEMs; values for 0–6 h and 0–24 h are adjusted means ± SEMs, with age used as a covariate. *n* = 14/group, except for RHI [*n* = 13 (EPs) and *n* = 14 (non-EPs)]; 1 participant was excluded due to incomplete occlusion during RHI assessment. Differences in study endpoints between interventions were analyzed by using ANOVA, with changes from baseline assessed by using a mixed general linear model including interaction between within-subject factor (repeated-measures ANOVA for treatment) and 1 between-subject factor (EP phenotype). Bonferroni post hoc corrections were conducted where significant between-group differences were identified. Changes were considered significant at *P* < 0.05. AI, augmentation index; BP, blood pressure; CO, cardiac output; EP, equol producer; RHI, reactive hyperemia index.

Although plasma equol concentrations in non-EPs reached a mean of 3.2 μmol/L after intervention with commercially produced S-(–)equol supplements (SE5-OH) (*P* < 0.001; non-EPs: 3.22 ± 0.47 μmol/L at 2 h), vascular function was not significantly changed at +2 h (**Table 4**). For data regarding the plasma isoflavone concentrations at 0 and 2 h after intake of commercially produced equol and the plasma isoflavone concentrations at 0, 6, and 24 h after the intake of isoflavones and placebo in EPs and non-EPs, see **Supplemental Tables 1**,** 2**, and **3** respectively.

**TABLE 4:** Hemodynamic and vascular measures at baseline and 2 h after non-EPs consumed a 40-mg equol supplement (SE5-OH) and EPs consumed a matched placebo (containing CMC)^1^

	EP phenotype				
	Non-EPs		EPs		*P*
	0 h	0–2 h	0 h	0–2 h	
RHI	2.78 ± 0.14	0.40 ± 0.23	2.71 ± 0.17	−0.09 ± 0.23	0.16
Diastolic BP, mm Hg	80 ± 2	1 ± 2	78 ± 2	0 ± 2	0.76
Systolic BP, mm Hg	134 ± 3	1 ± 2	125 ± 4	1 ± 2	0.94
CO, L/min	4.60 ± 0.25	−0.17 ± 0.17	3.93 ± 0.27	0.03 ± 0.17	0.44
AI, %	24 ± 1	0 ± 1	24 ± 1	−1 ± 1	0.19
cfPWV, m/s	9.8 ± 0.3	0.1 ± 0.2	10.2 ± 0.4	−0.5 ± 0.2	0.13

1 Baseline values are means ± SEMs; values for 0–2 h are adjusted means ± SEMs, with age used as a covariate. *n* = 14/group. Differences in study endpoints between interventions were analyzed by using general linear model (univariate analysis). AI, augmentation index; BP, blood pressure; cfPWV, carotid-femoral pulse-wave velocity; CMC, carboxy-methyl cellulose; CO, cardiac output; EP, equol producer; RHI, reactive hyperemia index.

## DISCUSSION

In one of the first randomized controlled isoflavone trials to prospectively recruit EPs to our knowledge, we observed acute improvements in cfPWV after soy isoflavone intakes at a dose readily achieved through the habitual diet and provide evidence of the potential significant importance of the EP phenotype. The potential clinical importance of this finding is noteworthy given that cfPWV, a measure of arterial stiffness, has been recently identified within the highest category of surrogate markers of clinical endpoints (38) and was previously identified as an independent predictor of vascular clinical outcomes (39). On the basis of previous data, which suggest a 14–15% increase in CVD events and all-cause mortality for each 1-m/s increment in cfPWV (40), our results would translate to an 11–12% risk reduction for middle-aged men at moderate risk of CVD, if sustained. Of fundamental importance, cfPWV improved only in those with the EP phenotype when there was a significant elevation in circulating equol concentration (i.e., at 24 h); EP status conferred no vascular benefits in the absence of equol (i.e., at 6 h), despite significant increases in circulating concentrations of the isoflavone precursors daidzein, genistein, and glycitein. These data support the in vitro evidence that shows equol to have more vasodilator capacity than its precursor, daidzein (12, 13). Previous studies have suggested that phosphorylation of endothelial NO synthase, which subsequently increases the bioavailability of NO, may be an important underlying mechanism for the vascular bioactivity of equol (34, 35, 41); in particular, acute inhibition of endothelial NO synthase has been shown to significantly increase pulse-wave velocity, independent of BP changes (42), which suggests the capacity for changes in NO concentrations to regulate key vascular function, without affecting BP. However, in our data set, we observed no effect of acute isoflavone supplementation on total NO (NO_2_^−^ + NO_3_^−^), suggesting either that chronic exposure to equol may be required to modify NO production or metabolism or that other previously reported mediators of arterial stiffness, such as vasoconstrictors (endothelin), inflammation, oxidative stress, or the function of smooth muscle cells (43), may be responsible for the acute effect we observed. Although there are currently insufficient data to determine the effect of chronic equol exposure on NO production, metabolism, and regulation, recent in vitro data support an effect of equol on smooth muscle function (44).

Although we showed an improvement in cfPWV, an effect on BP and endothelial peripheral arterial tonometry (measured by using EndoPAT) was not observed in our acute study. Although our findings are discrepant with previous cross-sectional or retrospective analyses (3, 4, 7, 14, 15), our acute data confirm the lack of effects on BP and flow-mediated dilation, which were recently observed in prospectively recruited postmenopausal EPs who consumed a daidzein-rich supplement for 6 mo (17). Taken together, our data and those of Liu et al. emphasize the importance of confirming the effects of the EP phenotype in prospectively recruited populations.

Interestingly, when we provided a commercially produced S-(–)equol supplement (SE5-OH) at a dose of 40 mg S-(–)equol to non-EPs we achieved a mean circulating equol concentration in excess of other studies that provided SE5-OH [3.2 μmol/L in our study compared with 1.42 μmol/L previously reported after the intake of 30 mg S-(–)equol (31)]; however, circulating concentrations in our male population were still below concentrations previously reported for postmenopausal women (4.95 μmol/L) (8). Despite successfully elevating circulating equol concentrations, we observed no significant vascular benefits 2 h after the intake of commercially produced equol supplements [a time chosen to coincide with peak plasma concentrations (8, 31)]. These findings imply that, at least in the acute setting, the EP phenotype is a necessity to unlock the vascular benefits of elevated equol concentrations. These findings are generally consistent with a previous 12-wk intervention with SE5-OH in which male EPs and non-EPs experienced no significant vascular improvements (21); however, in the same study, female postmenopausal non-EPs significantly reduced cardio-ankle vascular index (CAVI, a measure of aortic stiffness) after consumption of 10 mg S-(–)equol/d (21), suggesting potential differential effects by sex.

A strength of our trial is that, to our knowledge, it is one of the only studies to date that has prospectively recruited EPs, rather than using a retrospective post hoc analysis; the latter approach often has insufficient power to establish EP status by treatment effects (as a result of having unmatched EP and non-EP groups). In addition, our assessment of vascular function at times that coincide with the peak plasma concentration (6 and 24 h after intake) of the isoflavone precursor and metabolite (equol) (4, 8, 27, 45, 46) allowed us to confirm that the effects on cfPWV only coincided with significantly elevated equol concentrations, and not isoflavone parent compounds; these data support earlier in vitro data on the greater bioactivity of equol on vascular tissue response (i.e., cerebral artery) (12) and vascular smooth muscle response (13) than its precursor, daidzein. A potential limitation is that we only recruited men to our study, and therefore could not further investigate the potential sex disparities between EPs and vascular function observed in previous studies (21). Furthermore, future studies would benefit from extended assessments of vascular function (beyond 24 h) on the acute effects of SE5-OH on EPs and non-EPs using a crossover analysis design and on the sustained exposure of EPs to isoflavones. In combination, such studies would further elucidate the association between circulating equol concentrations and vascular function.

Overall, this trial, which prospectively recruited EPs and non-EPs, has provided evidence of the importance of the EP phenotype in mediating the favorable effects of dietary isoflavones on vascular health. Importantly, in our data set, the EP phenotype did not confer vascular benefits in the absence of equol, suggesting that dietary or alternative means of enhancing the microbial production of equol are essential for EPs to gain vascular benefits. Further data from long-term clinical trials that prospectively recruit EPs are required to confirm whether the acute improvements in cfPWV (which would equate to an 11–12% risk reduction for CVD) are sustained and of long-term clinical relevance.

## Footnotes

4 Abbreviations used: BP, blood pressure; cfPWV, carotid-femoral pulse-wave velocity; CVD, cardiovascular disease; EP, equol producer; MS/MS, tandem mass spectrometry; NO, nitric oxide; NO_2_^−^, nitrite; NO_3_^−^, nitrate.
